# Age-Related Macular Degeneration: New Insights in Diagnosis, Treatment, and Prevention

**DOI:** 10.3390/jcm11041064

**Published:** 2022-02-18

**Authors:** Thibaud Mathis, Laurent Kodjikian

**Affiliations:** 1 Service d’Ophtalmologie, Hôpital de la Croix-Rousse, Hospices Civils de Lyon, 69004 Lyon, France; thibaud.mathis@chu-lyon.fr; 2 UMR CNRS 5510 MATEIS, Université Lyon 1, 69100 Villeurbanne, France

Age-related macular degeneration (AMD) is an aging-related ocular disease that can be responsible for severe loss of visual acuity and loss of autonomy in patients. Although there has been a dramatic reduction in legal blindness resulting from AMD since the introduction of anti-vascular growth factor (VEGF) therapy in 2006 [1], the disease still causes a number of patients to progressively and irrevocably lose their vision despite treatment. However, the advent of the first approved intravitreal therapy for AMD has revolutionized AMD management, as well as our understanding of its pathophysiology and risk factors [2,3]. These findings have contributed to the emergence of major technological innovations in ophthalmology in recent years, particularly in terms of retinal imaging techniques. Multimodal examination now means very early, and extremely precise diagnoses can be made. From a therapeutic point of view, extensive research has been undertaken to investigate new molecules for the treatment of neovascular AMD developed in phases 1, 2, or 3. Atrophic AMD, which could be seen as the poor relation to the neovascular form, has also benefited from more research in these areas. This Special Issue on AMD is divided into three sections: (1) new insights into diagnosis and pathophysiology revealed by modern multimodal imaging; (2) current knowledge regarding treatment to improve visual outcomes, and the future therapeutic molecules being developed; (3) preventive medicine to control risk factors and limit vision loss.

The advent of multimodal imaging in ophthalmology has not simply revolutionized the diagnosis and management of retinal disease but has also led to advances in our understanding of the pathophysiology of the disease and the mechanisms leading to vision loss. The introduction of optical coherence tomography angiography (OCTA) was a great leap forward in terms of our ability to study the microvasculature of the macula and the changes brought about by AMD. Firstly, OCTA enables the noninvasive detection of the neovascular network by showing aberrant flow above or below the retinal pigment epithelium layer. Secondly, it can show modifications to the normal macular vascularization resulting from the disease itself or the aging of the retinal and choroidal tissue [4]. As demonstrated in diabetic macular edema, it appears that intraretinal fluid, which is associated with poorer visual prognosis in AMD, is related to worse vascular reperfusion when treated, in comparison to subretinal fluid [5]. Moreover, deep exploration inside the macular vascularization, at the choriocapillaris level, has demonstrated that the fellow-eyes in cases of unilateral neovascular AMD also showed vascular abnormalities such as nonexudative AMD (known as quiescent neovascularization, sometimes seen on invasive indocyanine green angiography), or choriocapillaris flow deficit, as already described in cases of high blood pressure or diabetes [4,6,7].

The earliest possible diagnosis of AMD and its neovascular complication allows for prompt treatment of the disease with intravitreal injections of anti-VEGF. Many studies have proved the efficacy and safety of bevacizumab, ranibizumab, and aflibercept in treating neovascular AMD [2,3,8] and other related diseases [9,10]. However, some differences in visual gain and anatomical outcomes exist between series, which appear to be associated with the baseline patient and disease characteristics and the treatment regimen used. Firstly, eyes presenting high visual acuity at baseline had a lower functional response despite maintaining higher final vision than other eyes [8,11]. Moreover, some other factors such as larger macular neovessels [12], increased age [8], or cardiovascular disease [13] were found to be independent risk factors of poor functional outcomes following anti-VEGF therapy. Secondly, the frequency of anti-VEGF injections seems to be one of the most relevant factors influencing functional outcomes [14]. This is supported by the results of randomized clinical trials (RCTs) that typically show better visual gain in comparison to real-life studies [8]. This is explained by the higher number of injections and better follow-up of patients in RCTs, thereby motivating frequent retreatment. Therefore, a proactive regimen usually leads to better outcomes in comparison to an as-needed regimen, as it maintains a high number of injections and reduced treatment burden with visits required. However, the follow-up of a patient with neovascular AMD treated under a proactive regimen is not always plain sailing and can bethrown off course by unforeseen events, such as the onset of another age-related disease. One example of this is the onset of cataracts, which contributes to vision loss. Although cataract surgery is a well-known risk factor for exudative recurrence, special care should be taken before surgery to controlling the neovascular process, and real-world data have suggested a minimum of 6 months of anti-VEGF treatment before surgery. Patients should also be advised of the uncertainty concerning the visual improvement they can expect following surgery [15]. Another example is the recurrence of neovascular exudation despite a short interval between two injections. The recurrence or persistence of retinal fluid in the long term, and macular thickness fluctuations, in particular, have been shown to be predictive factors of vision loss under treatment [16]. In order to minimize exudation, new therapeutic approaches have been developed or are currently under investigation. Brolucizumab is a novel 26 kDa single-chain antibody fragment targeting anti-VEGF and was approved by the FDA in 2019, following the completion of the HAWK and HARRIER RCTs [17]. Its small molecular size allows for a higher molecular concentration in a standard 0.05 mL volume dose. This results in an increased interval between injections in the RCTs and significantly improved control of exudation. The first real-world studies of brolucizumab for AMD seem to confirm these data [18,19], both in treatment-naïve eyes and when switching from another therapy [18,20]. However, the increased, although still rare, the occurrence of intraocular inflammatory adverse events (which can lead to vascular occlusion and vision loss) for this new molecule means a change in the treatment paradigm toward injections that are more spaced out is likely, helped by its improved efficacy [21]. In the coming years, the therapeutic arsenal will be totally transformed by numerous molecules in development [22], and the discovery of new therapeutic targets [23], with the aim of reducing the therapeutic burden and improving visual outcomes.

Generally speaking, the best way of preventing vision loss is to detect and diagnose the disease at an early stage, especially for the exudative form. Observational studies show that nearly one-fourth of patients are diagnosed and begin anti-VEGF treatment with visual acuity of 20/40 or better. However, it is well known that the patients with the best visual acuity at baseline retain better vision. It is therefore necessary to frequently monitor eyes with early or intermediate AMD. Nevertheless, requiring a large, aging population to attend clinics for frequent and regular ocular examinations brings with it its own set of difficulties. Identifying the patients at risk of disease progression at an early stage would be extremely helpful, as it means treatment can be initiated promptly, leading to better visual outcomes. A number of risk scores have been described, most of which include genetic polymorphism assessment and fundus examination [24,25,26]. However, they are not used in routine practice due to their complexity. The simplified test AMD risk-assessment scale (STARS^®^) is an easy-to-compile self-administered 13-item questionnaire evaluating individual risk for AMD in daily practice, focusing on demographic, cardiovascular, and lifestyle risk factors. A group of AMD specialists, using the Delphi method, has put forward recommendations to optimize the preventive care of patients in different geographical areas at risk of AMD based on STARS^®^ scoring. [27]. Another approach to preventing vision loss is to detect progression earlier by developing an at-home self-monitoring method. To date, only the Amsler grid is widely used in a number of different countries, but it has demonstrated poor sensitivity and specificity in detecting the disease at an early stage. Different self-monitoring devices have been tested, such as the hyperacuity test, to proactively monitor visual status and immediately alert the ophthalmologist in the event of a drop in vision. Although these tests have demonstrated good efficacy in clinical trials, and recent publications have begun to compile a growing body of evidence on their effectiveness, their use in clinical practice still needs to be studied further [28].

The diagnostic and therapeutic options currently available are only able to slow down the progression of macular degeneration and cannot stop it. Therefore, the identification of risk factors, which are known to contribute to disease progression, is critical, and many studies have focused on dietary habits, lifestyle, and light exposure. Although the association between smoking and AMD is widely accepted, the conclusions of studies evaluating the dietary intake of nutrients such as omega-3 fatty acids, beta carotene, lutein, and zeaxanthin seem less convincing. However, in the absence of new therapies capable of improving the clinical course of AMD, implementing preventive strategies could be an alternative, as well as advising a Mediterranean diet, which has also been shown to be effective in preventing the onset of AMD. Lastly, the role of light, ultraviolet rays, and blue light, in particular, needs to be further investigated since, to date, retinal toxicities associated with these wavelengths have only been demonstrated in experimental models [29].

In this Special Issue, we hope to review the basics and highlight the latest developments in AMD. To be sure, many other studies have been published elsewhere and have produced interesting data. This demonstrates the benefits of the international scientific community working on this disease, to limit its negative impacts, the most vital of which is the loss of visual function, leading to a loss of autonomy and a decrease in patients’ quality of life.
